# Biomimetic Cascade Polymer Nanoreactors for Starvation and Photodynamic Cancer Therapy

**DOI:** 10.3390/molecules26185609

**Published:** 2021-09-16

**Authors:** Shengda Liu, Tengfei Yan, Jianxin Sun, Fei Li, Jiayun Xu, Hongcheng Sun, Shuangjiang Yu, Junqiu Liu

**Affiliations:** 1 Key Laboratory of Organosilicon Chemistry and Material Technology, Ministry of Education, College of Material, Chemistry and Chemical Engineering, Hangzhou Normal University, Hangzhou 311121, China; liushengda0801@foxmail.com (S.L.); tengfeiyan@163.com (T.Y.); xujiayun@jlu.edu.cn (J.X.); sunhc@hznu.edu.cn (H.S.); 2 State Key Laboratory of Supramolecular Structure and Materials, College of Chemistry, Jilin University, Changchun 130012, China; jianxinsun2019@163.com (J.S.); sud716@163.com (F.L.)

**Keywords:** polymer nanocapsule, nanoreactor, starvation therapy, photodynamic therapy

## Abstract

The selective disruption of nutritional supplements and the metabolic routes of cancer cells offer a promising opportunity for more efficient cancer therapeutics. Herein, a biomimetic cascade polymer nanoreactor (GOx/CAT-NC) was fabricated by encapsulating glucose oxidase (GOx) and catalase (CAT) in a porphyrin polymer nanocapsule for combined starvation and photodynamic anticancer therapy. Internalized by cancer cells, the GOx/CAT-NCs facilitate microenvironmental oxidation by catalyzing endogenous H_2_O_2_ to form O_2_, thereby accelerating intracellular glucose catabolism and enhancing cytotoxic singlet oxygen (^1^O_2_) production with infrared irradiation. The GOx/CAT-NCs have demonstrated synergistic advantages in long-term starvation therapy and powerful photodynamic therapy (PDT) in cancer treatment, which inhibits tumor cells at more than twice the rate of starvation therapy alone. The biomimetic polymer nanoreactor will further contribute to the advancement of complementary modes of spatiotemporal control of cancer therapy.

## 1. Introduction

The alteration of cancer metabolic routes is a fascinating therapeutic approach to cancer treatment [1,2,3,4,5]. In general, the abnormal multiplication of cancer cells requires an adequate supply of nutrients and energies to sustain their existence and growth [6]. As a result, there is a rise in intracellular aerobic glycolysis, which is termed the Warburg effect, contributing to a greater sensitivity of cancer cells to alterations in glucose concentration [7,8,9]. Once glucose supply is interrupted, tumor growth is preferentially inhibited; thus, glucose metabolism-related cancer starvation therapy is increasingly regarded as a promising clinical translational approach [10,11,12,13]. To date, several strategies have been proposed to starve tumors by depleting glucose in cancer cells. In particular, the utilization of GOx to catalyze glucose metabolism is a proven effective anticancer strategy [14,15,16,17]. However, the upregulation of the adaptation of parallel energy supply to cancer cells may even result in a failure of starvation therapy. To extend the therapeutic effect, a complementary mode that cuts off the glucose supply to the cancer and destroys the cellular components associated with glucose metabolism was proposed.

As a complementary mode for synergistic therapy, PDT is a noninvasive method to precisely ablate local cancers through the tremendous oxidative power of reactive oxygen species (ROS) [18,19]. However, it is often necessary to convert the photosensitizers (PSs) into pharmaceutical formulations on account of their limited solubility [20,21]. In addition, the poor loading efficiency and the prior leakage behavior will further restrict their applications [22]. In recent years, covalent polymer nanocapsules with PSs as building blocks have been constructed for PDT, which demonstrate the advantages of easy diffusion of ^1^O_2_, avoidance of self-quenching, and high loading of PSs [23,24,25]. Several approaches are available for the preparation of covalent polymer nanocapsules, among which enzyme-catalyzed methods not only have the advantages of high efficiency, stability, and substrate specificity, but also allow the preparation of nanocapsules under mild conditions, which is very convenient to encapsulate natural enzymes [26].

**Figure 1:**
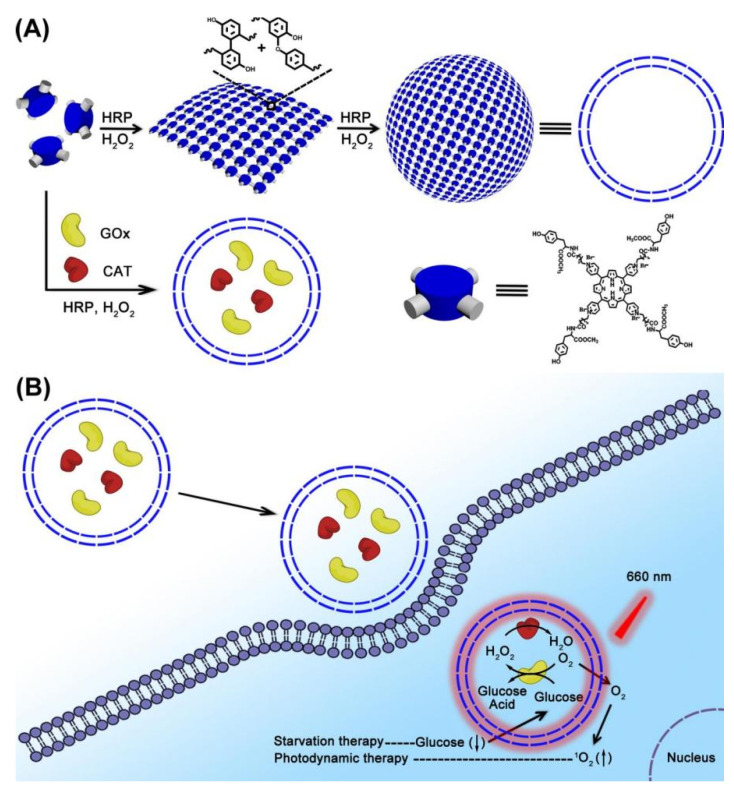
(**A**) The formation of nanocapsules and GOx/CAT-NCs. (**B**) The synergistic effects of GOx/CAT-NCs for starvation therapy and PDT under infrared irradiation.

As we know, covalent dimerization of tyrosine in a horseradish peroxidase (HRP)-catalyzed system was an efficient method for enzyme-catalyzed preparation of polymers [27,28,29]. Herein, polymer nanocapsules with porphyrin PSs as building blocks were constructed based on HRP-catalyzed tyrosine dimerization. Then, polymer nanocapsules further encapsulated GOx and CAT to obtain a biomimetic cascade nanoreactor (GOx/CAT-NC) (Figure 1). Benefiting from the enhanced permeability and retention (EPR) effect, GOx/CAT-NCs would be selectively internalized into cancer cells. The cascade reaction of GOx/CAT-NCs promoted O_2_ production catalyzed by endogenous H_2_O_2_, therefore speeding up glucose catabolism and accelerating cytotoxic ^1^O_2_ production under infrared irradiation, according to the following equations:

(1) $$H_{2}O_{2}\;\overset{\mathrm{CAT}}{\to }\;H_{2}O+O_{2}$$

(2) $$\mathrm{Glucose}+O_{2}\;\overset{\mathrm{GOx}}{\to }\;H_{2}O_{2}+\mathrm{Glucose}\;\mathrm{Acid}$$

(3) $$O_{2}\;\overset{\mathrm{GOX}/\mathrm{CAT}-\mathrm{NC}}{\underset{660\mathrm{nm}}{\to }}{\;}^{1}O_{2}$$

Here, CAT could regulate the hypoxic microenvironment of cancer by the catalytic degradation of endogenous H_2_O_2_. The generated O_2_ not only enhanced the glucose catabolism by GOx, strengthening the effect of starvation therapy, but also promoted the production of cytotoxic ^1^O_2_, improving the effect of PDT. Therefore, the combined effects of long-term starvation therapy and powerful PDT in cancer treatment would effectively suppress cancer growth in a spatiotemporally regulated manner.

## 2. Results and Discussion

### 2.1. Characterization of Polymer Nanocapsules

**Figure 2:**
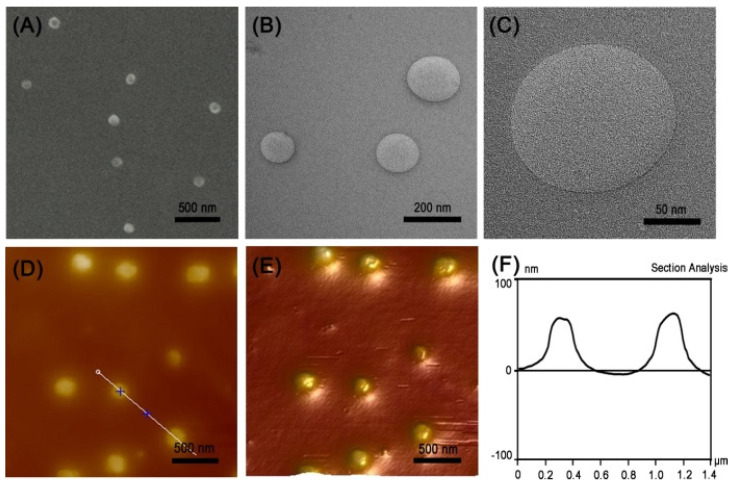
(**A**) SEM image, (**B**,**C**) TEM images, (**D**) AFM image, (**E**) 3D AFM image of the nanocapsules, and (**F**) associated height curve along the black line in panel D.

Polymer nanocapsules were prepared by an enzyme-catalyzed polymerization method. In brief, polymer nanocapsules were prepared by the polymerization of porphyrin-based building blocks containing tyrosine upon HRP and H_2_O_2_ addition. According to the previous literature, tyrosine dimerization resulted in a characteristic fluorescence emission peak at 410 nm [30,31,32]. Therefore, we first tested the fluorescence intensity of the system before and after the reaction. As shown in Figure S7, a fluorescence emission peak was observed at 410 nm after polymerization; therefore, the polymers were obtained by tyrosine dimerization. Then, the structure, morphology and dimension of the polymer nanocapsules were characterized by dynamic light scattering (DLS), scanning electron microscopy (SEM), transmission electron microscopy (TEM), and atomic force microscopy (AFM). According to the DLS results, the hydrodynamic diameter of the nanocapsules was 140 ± 60 nm (Figure S8). The Zeta potential results showed the nanocapsules were positively charged with 12.8 mV, which was suitable for penetrating into the cancer cells (Figure S9). As shown in Figure 2A, we could observe the spherical structure with uniform size by SEM images, and the size of which was basically consistent with the DLS results. Moreover, we had a direct view of the nanocapsules with hollow structures and thin walls by TEM (Figure 2B). The high-resolution TEM images clearly demonstrated the thin walls of nanospheres with the thickness of about 1 nm, which were probably monolayer thin walls (Figure 2C). The AFM images further confirmed that they were spherical structures with uniform height (Figure 2D–F).

### 2.2. Characterization of GOx/CAT-NCs

GOx/CAT-NCs were obtained by in situ encapsulation of GOx and CAT in polymer nanocapsules. The encapsulation of CAT and GOx by polymer nanocapsules was first investigated. In the experiments, coumarin-6-carboxylic acid was modified on the CAT surface to obtain CAT-coumarin, and fluorescein isothiocyanate was modified on the GOx surface to acquire GOx-FITC, where the small molecules unreacted to the enzyme surface were removed by dialysis. CAT-coumarin had an excitation wavelength of 325 nm and an emission wavelength of 460 nm with blue fluorescence, while GOx-FITC showed an excitation wavelength of 495 nm and an emission wavelength of 517 nm with green fluorescence (Figure S10). Then, we encapsulated CAT-coumarin and GOx-FITC in polymer nanocapsules, and the unencapsulated enzymes were removed by ultrafiltration and centrifugation. As shown in Figure S11, we could observe the nanocapsules with blue and green fluorescence by fluorescence microscopy images, proving that both enzymes were encapsulated in the nanocapsules. After successful encapsulation of the two enzymes by nanocapsules, we tested the encapsulation efficiency of the two enzymes by the standard curve of bovine serum albumin (BSA) using Coomassie Brilliant Blue method. The result showed that the encapsulation efficiency of the enzymes was 53.1%.

### 2.3. Properties of Polymer Nanocapsules and GOx/CAT-NCs

**Figure 3:**
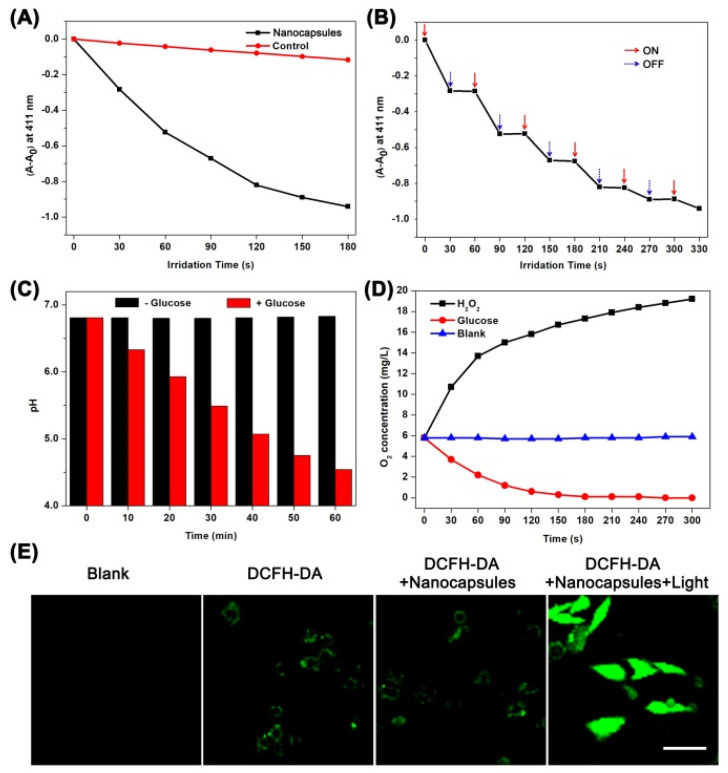
(**A**) The DPBF UV changes in the absence and presence of nanocapsules under infrared irradiation. (**B**) The DPBF UV changes in response of OFF-ON irradiation. (**C**) The pH value changes of GOx/CAT-NCs solution in the absence and presence of glucose. (**D**) The O_2_ concentration changes of GOx/CAT-NCs solution in the presence of H_2_O_2_ or glucose. (**E**) The CLSM images of 3T3 cells with various treatments. The scale bar is 20 μm.

Firstly, we measured the ^1^O_2_ production capacity of polymer nanocapsules by using the ^1^O_2_ sensor 1, 3-diphenylisobenzofuran (DPBF) as an indicator [33,34]. The UV absorption value at 411 nm of DPBF alone decreased weakly under infrared irradiation. However, with the addition of polymer nanocapsules, the UV absorption value at 411 nm decreased sharply when the mixture was irradiated with the same power of infrared light, and the UV absorption value decreased by 0.941 within 180 s (Figure 3A). More specifically, the nanocapsules producing ^1^O_2_ showed an accurate response to the illuminated state and the non-illuminated state (Figure 3B). In addition, we used 2′,7′-dichlorofluorescin diacetate (DCFH-DA) as a probe to determine the ability of polymer nanocapsules to generate ROS in real cells [35,36]. The 3T3 cells were pretreated with polymer nanocapsules and then treated with DCFH-DA fluorescent probe treatment to detect intracellular ROS levels. Whether the cells were co-incubated with single DCFH-DA or with polymer nanocapsules and DCFH-DA, we could only observe very weak fluorescence. While the cells co-incubated with DCFH-DA and polymer nanocapsules were irradiated with infrared light, we observed extremely strong fluorescence (Figure 3E). The results demonstrated that ROS could be generated in real cells by porphyrin-based polymer nanocapsules under infrared irradiation.

Furthermore, the catalytic ability of GOx/CAT-NCs was studied. As shown in Figure 3C, the pH value of the GOx/CAT-NCs solution without glucose treatment was maintained at about 6.81. In contrast, the addition of glucose to the GOx/CAT-NCs solution resulted in a dramatic decrease of the solution pH value (from 6.81 to 4.54) due to the catabolism of glucose by GOx and the generation of gluconic acid. Moreover, the dissolved oxygen content in the solution was monitored with the addition of glucose or H_2_O_2_ to the GOx/CAT-NCs solution, respectively. The dissolved oxygen content of the GOx/CAT-NCs solution alone was very stable at 5.8 mg L^−1^. When H_2_O_2_ was added, the dissolved oxygen content of the solution increased in a short time, rising to 19.2 mg L^−1^ within 300 s, because the CAT in the nanocapsules could decompose H_2_O_2_ into O_2_. When glucose was added, the dissolved oxygen content of the solution decreased shortly, dropping to 0 mg L^−1^ within 300 s, because of a process of oxygen consumption by the decomposition of glucose by the GOx in the nanocapsules (Figure 3D). The above results indicated that GOx/CAT-NCs had the catalytic activity of both CAT and GOx.

### 2.4. In Vitro Anticancer Effects of GOx/CAT-NCs

**Figure 4:**
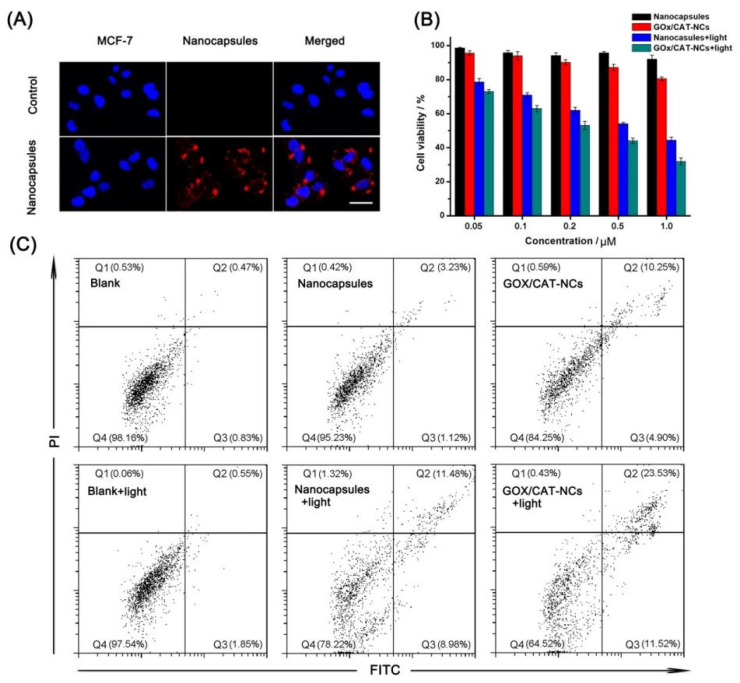
(**A**) The CLSM images of untreated MCF-7 cells and MCF-7 cells after incubation with nanocapsules, cell nuclei stained with Hoechst (blue channel). The scale bar is 50 μm. (**B**) MTT assay of the MCF-7 cells with various treatments. (**C**) Flow cytometry analysis of the MCF-7 cells with various treatments.

In order to investigate the uptake of the polymer nanocapsules by the cancer cells, we monitored the cell uptake process by using confocal laser microscopy (CLSM). As observed in Figure 4A, when the polymer nanocapsules were co-cultured with MCF-7 cells, red fluorescence belonging to the polymer nanocapsules was found in the cytoplasm of MCF-7 cells, indicating that the polymer nanocapsules could enter the cells by endocytosis. A prerequisite for polymer nanocapsules as carriers to be anticancer was that they were non-toxic to cells. Therefore, the toxicity of the nanocapsules was examined by 3-(4, 5-dimethylthiazol-2-yl)-2, 5-diphenyltetrazolium bromide (MTT) assay. After different concentrations of nanocapsules were co-cultured with cells, the cell survival rate was above 90.0%, which showed that the nanocapsules at low concentrations were almost non-toxic to cells (Figure 4B). Then, the cytotoxicity of polymer nanocapsules, GOx/CAT-NCs, polymer nanocapsules under infrared irradiation and GOx/CAT-NCs under infrared irradiation was measured, respectively. In all experiments, polymer nanocapsules and GOx/CAT-NCs at different concentrations were co-cultured with MCF-7 cells. In the experiments of PDT and synergistic treatment of starvation and photodynamics, the controls were exposed to infrared light for an additional 5 min. The cell survival rate reduced to 80.5%, when GOx/CAT-NCs (1 μM) were co-cultured with MCF-7 cells without infrared irradiation for starvation therapy alone. The cell survival rate reduced to 44.4%, when polymer nanocapsules (1 μM) were co-cultured with MCF-7 cells after infrared irradiation for PDT alone. After infrared irradiation, GOx/CAT-NCs (1 μM) co-cultured with MCF-7 cells reached a minimum cell survival rate of 31.9%, suggesting that starvation therapy and PDT in the system had a synergistic effect on cancer treatment (Figure 4B). Finally, we also used flow cytometry to determine apoptosis rates during starvation treatment, photodynamic treatment and synergistic treatment with starvation and photodynamics. The total mortality and apoptosis rates were 1.8%, 4.8%, and 15.8% for control MCF-7 cells, MCF-7 cells co-incubated with polymer nanocapsules and MCF-7 cells co-incubated with GOx/CAT-NCs, respectively, which were not exposed to infrared light. When exposed to infrared light, the MCF-7 cells alone had a combined mortality and apoptosis rate of 2.5%, which was almost unchanged. After infrared irradiation, MCF-7 cells co-cultured with polymer nanocapsules showed a rapid increase to the combined mortality and apoptosis rate of 21.8%. When co-culturing the MCF-7 cells with the GOx/CAT-NCs under infrared irradiation, the combined mortality and apoptosis rate rose sharply and increased to 35.5%, which showed the highest cell mortality and apoptosis rate and best treatment effect (Figure 4C). The in vitro results further demonstrated the distinct advantages of GOx/CAT-NCs in synergistic cancer therapy compared to single starvation therapy or PDT.

## 3. Experimental Section

### 3.1. Synthesis of Porphyrin-Based Building Block

The 4-pyridinecarboxaldehyde (30.0 mmol) and pyrrole (30.0 mmol) were added to 150.0 mL propionic acid at reflux for 3 h. After stopping heating and cooling to room temperature, the propionic acid solvent was removed by distillation under reduced pressure. Compound **1** was obtained by multiple recrystallization of the precipitate in ethanol, with a yield of 23%. ^1^H NMR (500 MHz, CDCl_3_, 25 °C) δ (ppm): δ 9.07 (d, 8H), 8.86 (s, 8H), 8.17 (d, 8H), −2.75 (s, 2H). ESI-MS: *m*/*z* 619.2 ([M + H]^+^).

Compound **1** (0.32 mmol) and 6-bromohexanoic acid (12.80 mmol) were placed in a flask and refluxed for 24 h with 80.0 mL *N*,*N*-dimethylformamide (DMF). After cooling to room temperature, the crude product was obtained by filtering and collecting the filter cake. Compound **2** was obtained by repeatedly washing the filter cake with chloroform and finally collecting the cake, with a yield of 41%. ^1^H NMR (500 MHz, DMSO, 25 °C) δ (ppm): δ 12.16 (s, 4H), 9.62 (d, 8H), 9.26 (s, 8H), 9.05 (d, 8H), 4.99 (m, 8H), 2.40 (m, 8H), 2.35–2.28 (m, 8H), 1.79–1.73 (m, 8H), 1.67–1.60 (m, 8H), −3.08 (s, 2H). ESI-MS: *m*/*z* 1075.5 ([M − 4Br − 3H]^+^).

Compound **2** (0.30 mmol), methyl L-tyrosinate hydrochloride (1.44 mmol) and benzotriazol-1-yloxytripyrroli-dinophosphonium hexafluorophosphate (PyBOP) (1.44 mmol) were placed in a flask and reacted with 10.0 mL anhydrous DMF and 1.0 mL anhydrous triethylamine (TEA) for 24 h at room temperature. After cooling to 4 °C and standing overnight, the crude product was obtained by filtering and collecting the filter cake. Compound **3** (porphyrin-based building block) was obtained by repeatedly washing the filter cake with methanol and finally collecting the filter cake, with a yield of 65%. ^1^H NMR (500 MHz, DMSO, 25 °C) δ (ppm): δ 9.54 (d, 8H), 9.36 (s, 4H), 9.22 (s, 4H), 9.01 (d, 8H), 8.33 (s, 4H), 7.05 (d, 8H) 6.67 (d, 8H), 4.86–5.00 (m, 8H), 4.40–4.52 (m, 4H), 3.60 (s, 12H), 2.76–3.00 (m, 8H), 2.18–2.30 (m, 16H), 1.62–1.74 (m, 8H), 1.44–1.56 (m, 8H), −3.08 (s, 2H). ESI-MS: *m*/*z* 1783.8 ([M − 4Br − 3H]^+^).

### 3.2. Preparation of Polymer Nanocapsules and GOx/CAT-NCs

Porphyrin-based building block was dissolved in DMF solvent and a solution of 10^−2^ M was prepared. To a total volume of 1 mL of aqueous system, 10 μL of the above DMF solution was added to form a 10^−4^ M aqueous solution of porphyrin-based building block. Then, 0.1 μg HRP and 10^−2^ M H_2_O_2_ were added to the system and the polymer nanocapsules were obtained after standing for 60 min.

Porphyrin-based building block was dissolved in DMF solvent and prepared as a 10^−2^ M solution. To a total volume of 1 mL of aqueous system, 10 μL of the above DMF solution was introduced to produce a 10^−4^ M aqueous solution of porphyrin-based building block. Then, it was added 10 μg CAT, 10 μg GOx, 0.1 μg HRP and 10^−2^ M H_2_O_2_ to the system and left for 60 min to obtain the mixture. Finally, the non-encapsulated CAT and GOx were removed by ultrafiltration centrifuge tubes (M_W_: D) to obtain GOx/CAT-NCs. 

### 3.3. ^1^O_2_ Production Ability of Polymer Nanocapsules

First, ^1^O_2_ production under irradiation was assessed. In the experiment, we added 10^−5^ M polymer nanocapsules and 50 μM DPBF to the system. In the control, we only added 50 μM of DPBF to the system without the addition of nanocapsules. Both groups were irradiated by 660 nm laser for the same time. Then, the response of OFF/ON irradiation to ^1^O_2_ generation was measured. The system was the same as that previously used in the experiment. We first irradiated the system with infrared light for 30 s to detect the products, and then stopped the irradiation for 30 s for further detection of the products, which was repeated five times. At last, the ROS generation in the cells was evaluated. The 3T3 cells were first cultured for 4 h. Subsequently, the old medium was replaced with new medium and new medium containing polymer nanocapsules, respectively. The cells continued to be cultured for 4 h. Then, the polymer nanocapsules that did not enter the cells were washed away by washing three times with phosphate buffer (pH = 7.4). After co-culture of nanocapsules and cells, the systems were exposed to irradiation by 660 nm laser for 5 min or none of irradiation, respectively. Next, DCFH-DA was added to the systems at 37 °C for 30 min. The systems were identified by CLSM. 

### 3.4. Catalytic Activity Determination of GOx/CAT-NCs

First, the pH change of GOx/CAT-NCs solution was measured without or with the addition of glucose. GOx/CAT-NCs (5 × 10^−6^ M) were mixed with glucose solution (50 mg mL^−^^1^) or distilled water, respectively. We measured the pH value of the solution in real time with a pH meter. Then, the dissolved oxygen content of GOx/CAT-NCs solution was measured with the addition of glucose or H_2_O_2_. The dissolved oxygen content in solution was monitored in real time by a dissolved oxygen meter when glucose (50 mg mL^−^^1^) or H_2_O_2_ (50 mM) was added to the solution containing in GOx/CAT-NCs (5 × 10^−6^ M).

### 3.5. Cell Culture and Cytotoxicity Assays

The cells used in the experiments were MCF-7 cells and 3T3 cells from the Institute of Biochemistry and Cell Biology, Shanghai Institute for Biological Sciences, Chinese Academy of Sciences (Shanghai, China). The cell culture medium was RPMI-1640 medium containing 10% FBS and 1% antibiotics. The cell culture environment was 37 °C with 5% CO_2_ in a cell culture incubator at 37 °C.

The toxicity of polymer nanocapsules or GOx/CAT-NCs on MCF-7 cells under different conditions was examined by MTT assay. First, the MCF-7 cells were incubated in a 96-well plate for 24 h. Then, the culture medium was removed, and different concentrations of polymer nanocapsules or GOx/CAT-NCs and the cells were co-incubated in new medium for 24 h. The controls were irradiated by 660 nm laser (100 mW cm^−^^2^) for another 5 min. All above systems were treated with MTT for 4 h. Insoluble formazan crystals were generated in the well plates after MTT treatment. Finally, the medium was carefully removed and dimethyl sulfoxide (DMSO) was added to the well plates. The cell viability could be determined by measuring the absorbance of the dissolved formazan at 492 nm.

The apoptosis rates were detected by flow cytometry. The MCF-7 cells were placed in 24-well plates and incubated with polymer nanocapsules (1 μM) or GOx/CAT-NCs (1 μM). The cells were incubated for 12 h, where the controls were irradiated by a 660 nm laser (100 mW cm^−^^2^) for another 5 min. Then, all above systems were treated with trypsin after incubation. After washing twice with PBS, the cells were stained with Annexin V-FITC/PI kit for 15 min and tested by flow cytometry.

## 4. Conclusions

In summary, a biomimetic cascade polymer nanoreactor, GOx/CAT-NC, was developed to improve the anticancer therapeutic efficiency with synergistic starvation and photodynamic therapy. The integration of starvation therapy and PDT was a safe cancer treatment strategy because of the ability to modulate the intracellular aerobic glycolysis and to photocontrol the toxicity of PDT in cancer cells. Furthermore, by transferring H_2_O_2_ to O_2_, the GOx/CAT-NCs could be applied to spatiotemporally controlled cancer therapy by reducing tumor hypoxia. Therefore, cutting off the glucose metabolism of cancer cells by long-term starvation therapy and damaging intracellular components by powerful PDT was a highly efficient strategy for synergistic anticancer. The biomimetic cascade nanoreactor will further contribute to the advancement of complementary modes to treat cancer more effectively despite the adverse cancer microenvironment.

## Data Availability

The data presented in this study are available upon request from the corresponding author.

## Supplementary Materials

The following are available online. Figure S1. ^1^H-NMR spectrum of compound **1**. Figure S2. ESI-MS analysis of compound **1**. Figure S3.^1^H-NMR spectrum of compound **2**. Figure S4. ESI-MS analysis of compound **2**. Figure S5. ^1^H-NMR spectrum of compound **3**. Figure S6. ESI-MS analysis of compound **3**. Figure S7. Fluorescence analysis of monomer and polymer nanocapsule. Figure S8. Hydrodynamic sizes of the nanocapsules in aqueous solution. Figure S9. Zeta protential distribution of the nanocapsules in aqueous solution. Figure S10. Fluorescence analysis of CAT-Coumarin and GOx-FITC. Figure S11. Fluorescence microscopy images of nanocapsules encapsulating CAT-Coumarin and GOx-FITC.
