# Supplementary material for: Biomimetic Cascade Polymer Nanoreactors for Starvation and Photodynamic Cancer Therapy
Source: Molecules. 2021 Sep 16;26(18):5609. doi: 10.3390/molecules26185609 (PMC8470963; doi:10.3390/molecules26185609)
Supplement: Supplementary file 1 [file molecules-26-05609-s001.zip › molecules-1378148-supplementary.pdf]

Biomimetic cascade polymer nanoreactors for starvation and photodynamic cancer therapy

Shengda Liu [1], Tengfei Yan [1], Jianxin Sun [2], Fei Li [2], Jiayun Xu [1], Hongcheng Sun [1], Shuangjiang Yu [1,*] and Junqiu Liu [1,*]

1 College of Material, Chemistry and Chemical Engineering, Key Laboratory of Organosilicon Chemistry and Material Technology, Ministry of Education, Hangzhou Normal University, Hangzhou 311121, China

2 State Key Laboratory of Supramolecular Structure and Materials, College of Chemistry, Jilin University, Changchun 130012, China

* Correspondence: yusj@hznu.edu.cn; junqiuliu@jlu.edu.cn

Materials

4-Pyridinecarboxaldehyde, pyrrole, 6-bromohexanoic acid, methyl L-tyrosinate hydrochloride were purchased from Energy Chemical. Triethylamine (TEA) and benzotriazol-1-yloxytripyrroli-dinophosphonium hexafluorophosphate (PyBOP) were purchased from Aladdin Biochemical Technology Co., Ltd. RPMI 1640 medium, fetal bovine serum (FBS), 3-(4,5-dimethylthiazol-2-yl)-2,5-diphenyltetrazolium bromide (MTT), Hoechst 33342 staining solution, trypsin and glucose oxidase (GOx) were purchased from Meilun Biotechnology Co., Ltd. Catalase (CAT) and horseradish peroxidase (HRP) were purchased in Sigma-Aldrich. Other chemical reagents were purchased from Beijing Chemical Works Co.,Ltd.

Synthesis of porphyrin-based building block (compound 3)

Compound 1 was produced by using 4-pyridinecarboxaldehyde and pyrrole. Then, compound 2 was prepared by modifying Compound 1 with 6-bromohexanoic acid. At last, compound 3 was obtained by modifying compound 2 with methyl L-tyrosinate hydrochloride. The synthesis route is shown in the following figure.

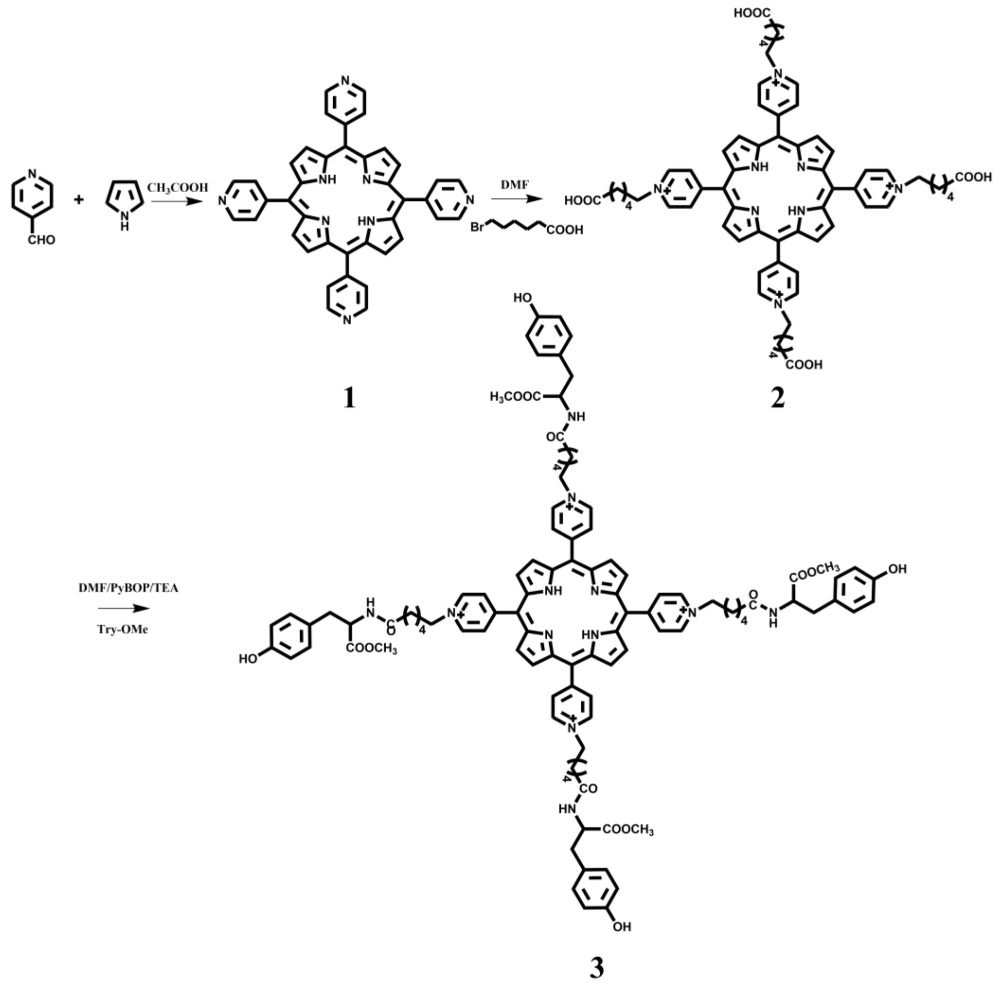

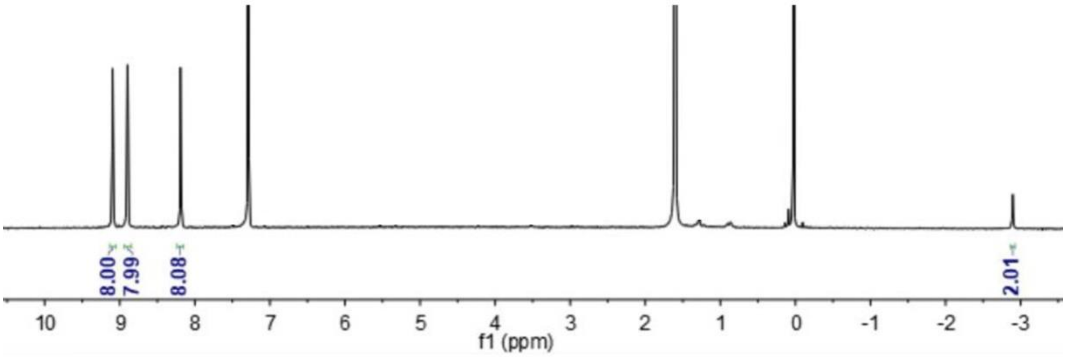

Figure S1 ^{1}H NMR spectrum of compound **1** in $CDCl_3$.

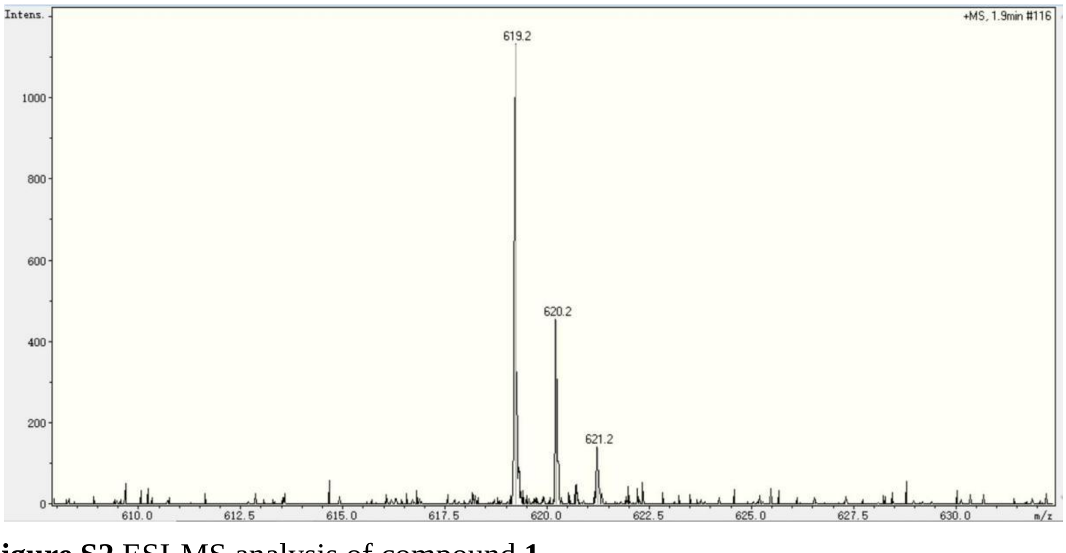

Figure S2 ESI-MS analysis of compound **1**.

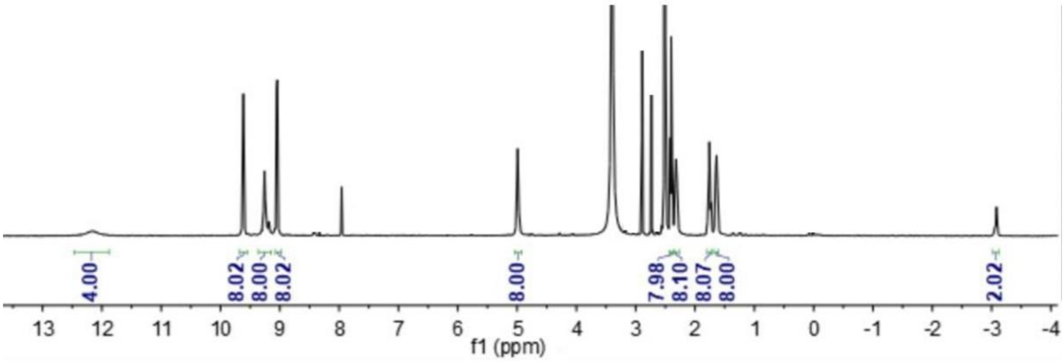

Figure S3 ^{1}H NMR spectrum of compound **2** in DMSO.

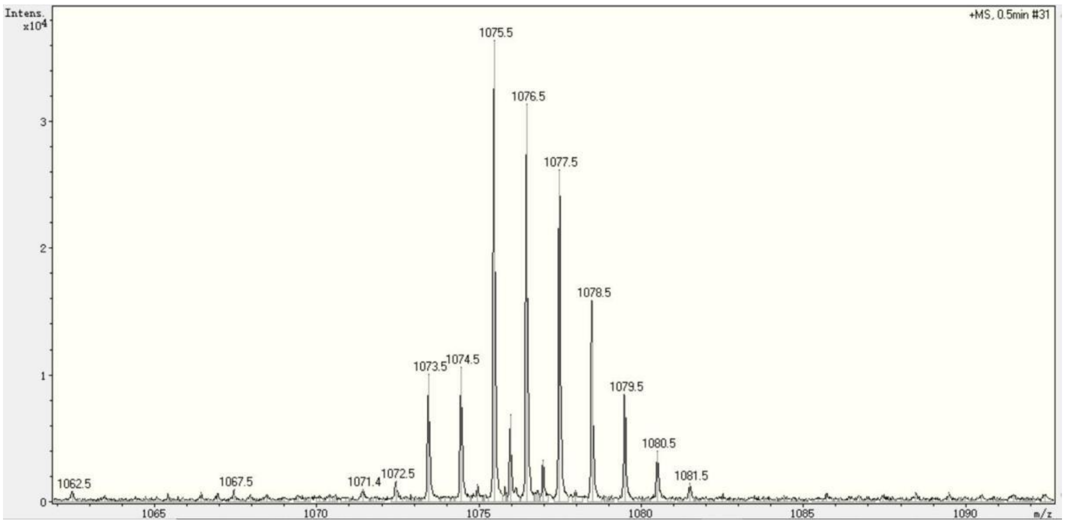

Figure S4 ESI-MS analysis of compound **2**.

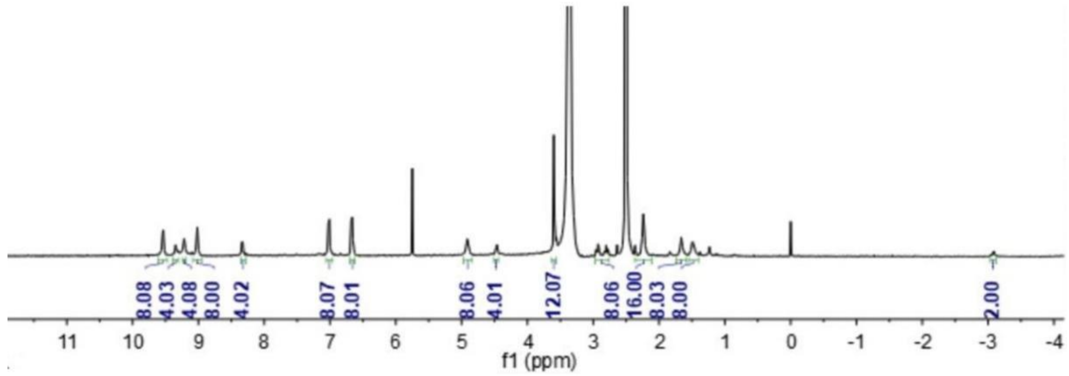

Figure S5 ^{1}H NMR spectrum of compound **3** in DMSO.

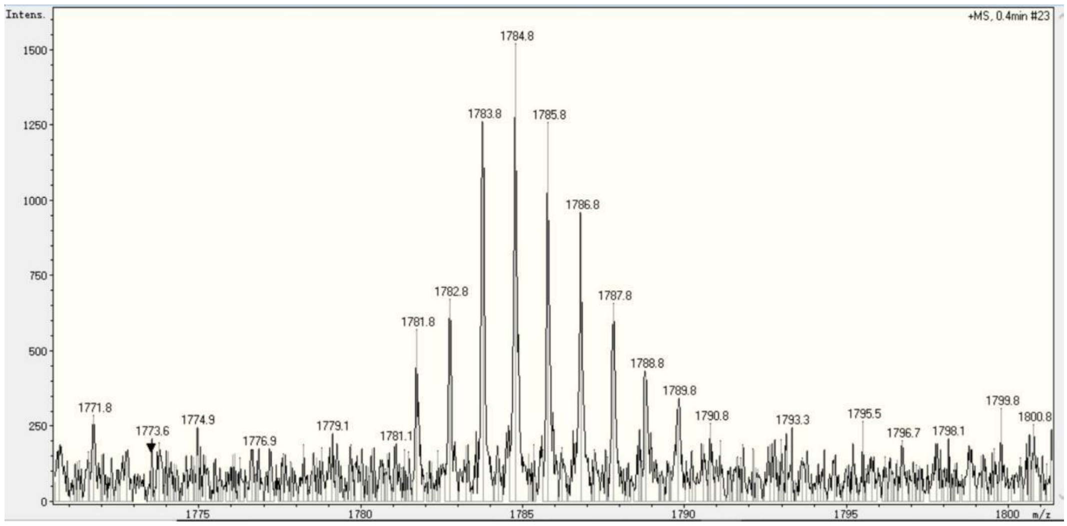

Figure S6 ESI-MS analysis of compound **3**.

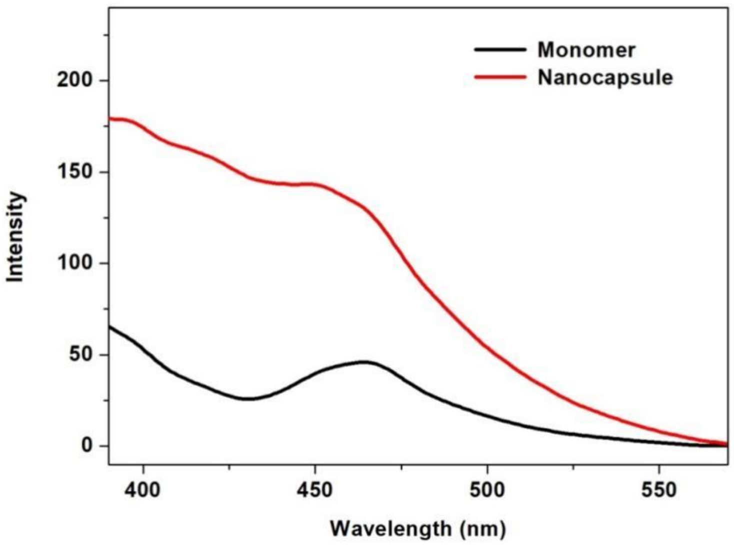

Figure S7 Fluorescence analysis of monomer and polymer nanocapsules.

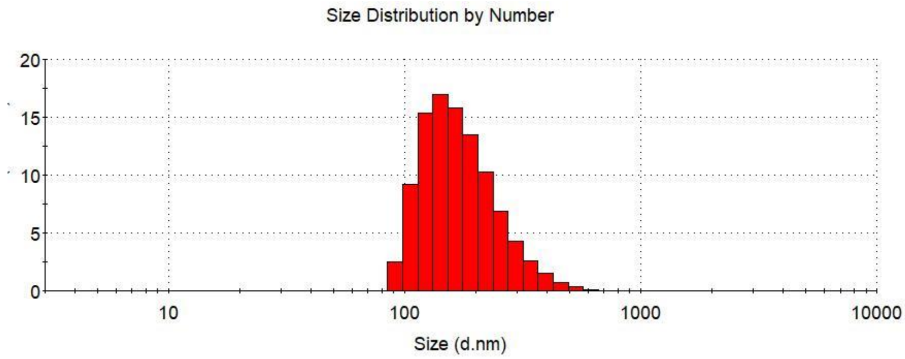

Figure S8 Hydrodynamic sizes of the nanocapsules in aqueous solution.

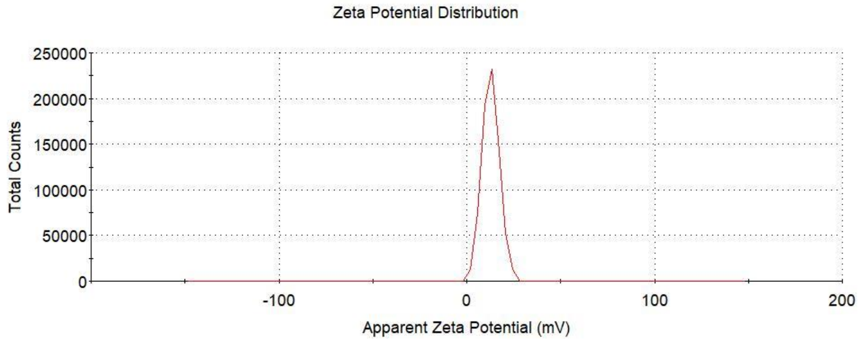

Figure S9 Zeta protential distribution of the nanocapsules in aqueous solution.

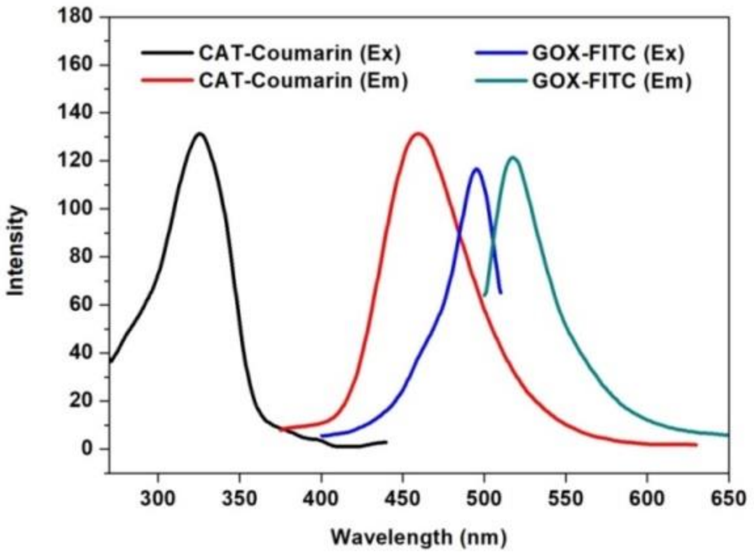

Figure S10 Fluorescence analysis of CAT-Coumarin and GOx-FITC..

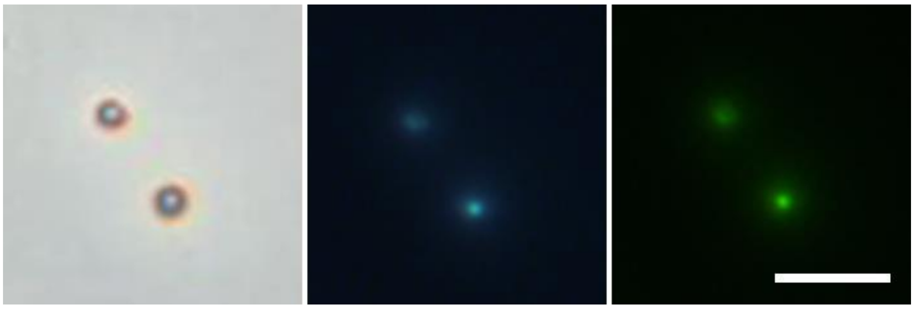

Figure S11 Fluorescence microscopy images of nanocapsules encapsulating CAT-Coumarin and GOx-FITC.
